# Differential Modulation of Markers of Oxidative Stress and DNA Damage in Arterial Hypertension

**DOI:** 10.3390/antiox12111965

**Published:** 2023-11-04

**Authors:** Moritz Kreutzmann, Bettina J. Kraus, Martin Christa, Stefan Störk, Eugène H. J. M. Jansen, Helga Stopper, Nicole Schupp

**Affiliations:** 1 Institute of Toxicology, Medical Faculty, University of Düsseldorf, 40225 Düsseldorf, Germany; kreutzmann.m@gmx.de; 2 Department of Clinical Research & Epidemiology, Comprehensive Heart Failure Centre, University Hospital Würzburg, 97078 Würzburg, Germanychrista_m@ukw.de (M.C.); stoerk_s@ukw.de (S.S.); 3 Department of Medicine I, University Hospital Würzburg, 97080 Würzburg, Germany; 4 Boehringer Ingelheim International GmbH, 55216 Ingelheim, Germany; 5 Centre for Health Protection, National Institute for Public Health and the Environment, 3721 MA Bilthoven, The Netherlands; eugenejansen99@gmail.com; 6 Institute of Pharmacology and Toxicology, University Hospital Würzburg, 97080 Würzburg, Germany; stopper@toxi.uni-wuerzburg.de

**Keywords:** high blood pressure, 3-nitrotyrosine, 8-oxodG, SHp, γ-H2AX

## Abstract

Patients with arterial hypertension have an increased risk of developing tumors, particularly renal cell carcinoma. Arterial hypertension is linked to DNA damage via the generation of oxidative stress, in which an upregulated renin–angiotensin–aldosterone system plays a crucial role. The current study investigated surrogates of oxidative stress and DNA damage in a group of hypertensive patients (HypAll, n = 64) and subgroups of well (HypWell, n = 36) and poorly (HypPoor, n = 28) controlled hypertensive patients compared to healthy controls (n = 8). In addition, a longitudinal analysis was performed with some of the hypertensive patients. Markers for oxidative stress in plasma (SHp, D-ROM, and 3-nitrotyrosine) and urine (8-oxodG, 15-F_2t_-isoprostane, and malondialdehyde) and markers for DNA damage in lymphocytes (γ-H2AX and micronuclei) were measured. In HypAll, all markers of oxidative stress except malondialdehyde were increased compared to the controls. After adjustment for age, this association was maintained for the protein stress markers SHp and 3-nitrotyrosine. With regard to the markers for DNA damage, there was no difference between HypAll and the controls. Further, no significant differences became apparent in the levels of both oxidative stress and DNA damage between HypWell and HypPoor. Finally, a positive correlation between the development of blood pressure and oxidative stress was observed in the longitudinal study based on the changes in D-ROM and systolic blood pressure. In conclusion, we found increased oxidative stress in extensively treated hypertensive patients correlating with the level of blood-pressure control but no association with DNA damage.

## 1. Introduction

In 2019, arterial hypertension affected approximately 33% of the global population over the age of 30 [1], with prevalence increasing with age. It is the most important cardiovascular risk factor and is responsible for about 10.9 million deaths annually [2]. Epidemiological studies have found an increased incidence of tumors among patients with arterial hypertension [3]. In particular, the risk of developing renal cell carcinoma is approximately doubled [3,4,5,6]. Arterial hypertension has also been identified as a risk factor in other cancer entities, such as endometrial [7], prostate [8], or breast carcinoma [9,10]. The elucidation of the biological mechanisms of the connection between arterial hypertension and tumor diseases is the subject of current investigations. The renin–angiotensin–aldosterone system (RAAS), which is a hormonal mechanism that regulates blood pressure, is thought to play an important role. Data from molecular, animal, and clinical studies suggest that an upregulated RAAS influences carcinogenesis and cancer growth, among other things, through the induction of oxidative stress [11].

Oxidative stress describes the state of a system in which oxidants are present in excess compared to antioxidants. This imbalance is the result of increased formation of reactive oxygen species (ROS) and/or reduced antioxidant defense mechanisms [12]. In elevated concentrations, ROS damage numerous biomolecules, contributing to the development of mainly age-related diseases, such as atherosclerosis, tumors, and neurodegeneration [13]. Crucial for the induction of tumors by oxidative stress are the DNA lesions caused by ROS. Over 100 oxidative DNA modifications are already known. These include single and double-strand breaks, DNA cross links, and changes in purine and pyrimidine bases and deoxyribose [14]. Chronically elevated ROS levels, which exceed the capacity of DNA repair mechanisms, lead to the accumulation of DNA damage. If this is not repaired, genomic instability occurs, which can lead to the development of carcinomas [15].

In rats, we demonstrated that elevation of blood pressure by angiotensin II infusion resulted in an increased mutation frequency in the kidney, likely due to oxidative stress induced by the hormone and subsequent oxidative DNA lesions [16]. In the present study, we analyzed the blood and urine of well and poorly controlled hypertensive patients. We targeted markers of oxidative stress and DNA damage to estimate systemic oxidative stress and DNA damage, considering their influence through antihypertensive therapy. One marker of DNA damage utilized here was the count of micronuclei (MN) in peripheral blood cells. MN consist of chromosome fragments surrounded by a nuclear envelope. The abundance of MN in blood cells was found to be predictive of cancer risk, especially of urogenital cancers, in a population of healthy subjects [17]. With the combination of markers of oxidative stress and DNA damage, we wanted to test the hypothesis that hypertensive patients have an increased oxidative burden and also higher basal DNA damage, which would fit into the observation of an increased risk of kidney cancer in this group of patients.

## 2. Materials and Methods

### 2.1. Patient Recruitment

Patients were recruited via the outpatient consultation for refractory hypertension at the University Hospital of Würzburg (UKW) and via the Myocardial Sodium Content In Cardiac Disease (MyStIC) study [18]. In the consultation for therapy-resistant hypertension, patients are treated who have a spontaneous systolic blood pressure >160 mmHg or an average blood pressure >135/85 mmHg in their ambulatory blood-pressure monitoring (ABPM) despite taking (originally) at least three antihypertensive drugs. As a baseline evaluation, secondary forms of hypertension were excluded. The data obtained in the consultation were entered into the Würzburg registry for therapy-resistant hypertension (Best-In-Tension Study) after providing written informed consent. Blood and urine samples for further testing for markers of oxidative stress and genome damage were collected during the consultation; clinical data were obtained from the registry. Blood samples were also obtained, and clinical parameters were determined in 16 and 11 of the enrolled patients, respectively, (visit 1 = V1), 7 ± 2 months (follow-up 1 = FUP 1), and 14 ± 2 months (follow-up 2 = FUP 2) after switching their previous antihypertensive therapy to new combination therapy, mainly by switching from cardio-selective to nonselective betablockers or from short- to long-acting RAAS inhibitors. 

The MyStIC study was a clinical study of the German Centre for Heart Failure (DZHI) of the UKW to measure the sodium content in the heart and skeletal muscle. It was an observational study using a sodium MRI to determine differences in sodium storage in three different groups: (1) patients with primary hyperaldosteronism, (2) patients with essential hypertension, and (3) healthy volunteers. The inclusion criteria for the Best-In-Tension Study registry and the inclusion and exclusion criteria of the MyStIC study are listed in Table 1.

Thus, patients with arterial hypertension were recruited from the Best-In-Tension registry and the MyStIC study, whereas healthy volunteers, as a control group, were recruited solely through MyStIC. Of the original 12 healthy volunteers, 2 subjects each (in total 4) had to be transferred from the control group to the HypWell and HypPoor groups because of detected high blood pressure. Blood samples and urine for further examination of markers of oxidative stress and genomic damage were taken as part of the baseline examination of the patients or subjects; the clinical data obtained for this purpose were obtained from the study database. Approval for the Best-In-Tension Study and the MyStIC Study was granted by the responsible ethics committee of the medical faculty of the University of Würzburg (No. 311/12 and No. 220/13, respectively).

### 2.2. Data Collection

The data collection of all patients and healthy subjects was carried out equally. Blood and urine parameters were determined by the central laboratory of the University Hospital Würzburg. The physical examination included the determination of body height and weight as well as the measurement of blood pressure on the upper arm using a standard sphygmomanometer. After the patient sat down, the blood-pressure cuff was applied, and 5 min of rest was observed. Three measurements were taken at intervals of 2 min each on the same arm according to the following scheme: 1st measurement in the sitting position, 2nd measurement in the lying position, and 3rd measurement while standing. Ambulatory blood-pressure monitoring was performed either at the DZHI or externally. Information on pre-existing conditions, cardiovascular risk factors, and current medication was obtained from the medical history and previous doctor’s notes. Most laboratory parameters were available from almost all participants, with the exception of some parameters: for example, 21 and 19 complete cases for proteinuria/albuminuria, 15 and 20 for ACE, and 25 and 24 for uric acid were available for HypWell (n = 36) and HypPoor (n = 28), respectively.

### 2.3. Blood Collection and Processing for Determination of Stress Parameters and DNA Damage

Peripheral venous blood was collected into a 9 mL EDTA monovette (Sarstedt, Nümbrecht, Germany). Blood separation by gradient centrifugation, as described by Schupp et al. [19], started within two hours after collection. The top layer, corresponding to the plasma, was removed with a Pasteur pipette to about 0.5 cm above the opaque interphase, aliquoted, and stored at −20 °C. The opaque layer containing lymphocytes and other mononuclear cells was also picked up with a Pasteur pipette and placed in a new 15 mL conical centrifuge tube, washed twice with RPMI 1640 medium (Sigma-Aldrich, Taufkirchen, Germany), and resuspended in 3 mL RPMI 1640 medium supplemented with 15% FCS, 1% L-glutamine, 1% Na-pyruvate, 1% MEM nonessential amino acid solution, and penicillin/streptomycin. After the determination of the cell count by means of a counting chamber (Neubauer improved, Hecht Assistent, Sondheim, Germany), the lymphocytes were further used for different experiments and divided accordingly.

### 2.4. Urine Processing for the Determination of Stress Parameters

The urine samples given by the test persons reached the laboratory in 10 mL monovettes within two hours and were centrifuged at 4 °C for 10 min at 4100× *g* (Universal 16R, Hettich, Tuttlingen, Germany). The sediment was discarded and the urine was aliquoted and stored at −20 °C.

### 2.5. γ-H2AX Staining

Immediately following PBMC isolation, 20,000 cells each were applied to precleaned slides using cytocentrifugation (Cytospin 3, Thermo Scientific, Braunschweig, Germany). The cells were fixed in ice-cold methanol for at least 24 h, washed with PBS, blocked with 5% donkey serum (Biochrom, Berlin, Germany), 1% BSA, and 0.3% Triton X-100 in PBS for 30 min, and then incubated 1 h at 37 °C with anti-gamma H2A.X (phospho S139) antibody (EP854(2)Y, ab81299, Abcam, Cambridge, UK) at a dilution of 1:100 in a blocking solution. After extensive washing with PBS containing 0.2% Tween, they were incubated for 30 min at 37 °C in the dark with a fluorescein isothiocyanate-conjugated secondary antibody (CF488A Donkey Antirabbit IgG (H + L), #20015, Biotium, Hayward, CA, USA). Nuclei were counterstained with DAPI, and the slides were mounted with Confocal Matrix (Micro Tech Lab, Graz, Austria). Images were captured using a Nikon Eclipse 55i fluorescence microscope (Nikon GmbH, Düsseldorf, Germany) at a 400-fold magnification. Quantification was done by counting γ-H2AX foci in 50 cells per individuum with ImageJ 1.47 v (http://rsb.info.nih.gov/ij/ (accessed on 24 June 2015) [20]) after blinding the samples.

### 2.6. Micronucleus Frequency Test

Immediately after isolation of the PBMC, 5 mL of the leucocyte suspension containing approximately 1 million cells/mL was transferred into a 25 cm^2^ cell culture flask (Greiner Bio-One, Kremsmünster, Austria). To stimulate mitosis of predominantly lymphocytes, 10 µg/mL phytohaemagglutinin was added. The cell culture flasks were then incubated standing at 37 °C and 5% CO_2_ for 24 h in an incubator (Heraeus, Hanau, Germany). The cells were centrifuged on pre-cleaned slides in ice-cold methanol. Staining was performed with the nucleic acid stain GelGreen (Biotium, Hayward, CA, USA) before mounting the slides with DABCO; 2000 cells with single-segmented, round to oval nuclei (characteristics of lymphocytes) per individuum were evaluated on the day of staining and after blinding, at a 400× magnification on the Eclipse 55i microscope (Nikon, Düsseldorf, Germany). On the one hand, the proportion of double-nucleated and multinucleated (≥3 nuclei) cells, as well as apoptoses and mitoses, in 2000 single-nucleated cells (EC) were counted and, on the other hand, nuclear abnormalities such as MN, nuclear buds, nuclear blebs, and NPBs were recorded. The latter three are shown combined as “nuclear anomalies (NA)”. The evaluation criteria were based on those of Fenech [21]. Deviating from the protocol of Fenech, cell division was not arrested by cytochalasin B in the anaphase; therefore, the abnormalities were counted in mononuclear lymphocytes.

### 2.7. Markers for Oxidative/Nitrosative Stress in Plasma

The plasma samples were used to detect three different markers for oxidative and nitrosative stress, respectively.

#### 2.7.1. D-ROM

The derivatives of reactive oxygen metabolites (D-ROM) test was used as a biomarker for oxidative stress. Since ROS have a very short half-life [22], the more stable D-ROMs are detected in plasma. Measurements were performed at the Centre for Health Protection, National Institute for Public Health and the Environment, Bilthoven, The Netherlands, using a D-ROM assay kit (Diacron, Grosseto, Italy) and the LX20-Pro autoanalyzer (Beckman-Coulter, Woerden, The Netherlands). The test result is expressed in CARR U (Carratelli Units), where 1 CARR U corresponds to 0.08 mg H_2_O_2_/100 mL plasma [23].

#### 2.7.2. SHp

The assay for thiol groups in proteins (SHp) is a photometric method for the determination of the thiol concentration in plasma and was performed at the Centre for Health Protection, National Institute for Public Health and the Environment, Bilthoven, The Netherlands, using the SHp test (Diacron, Grosseto, Italy) as described in Jansen et al. [24].

#### 2.7.3. 3-Nitrotyrosine

As a marker of nitrosative stress, 3-nitrotyrosine in plasma was measured with the Nitrotyrosine ELISA Kit (StressMarq Biosciences Inc., Victoria, BC, Canada) according to the protocol provided by the manufacturer.

### 2.8. Markers for Oxidative Stress in Urine

Three different markers of oxidative stress were detected in spontaneous urine, and each was related to creatinine concentration.

15-F_2t_-isoprostane was measured using the “Urinary Isoprostane EIA Kit (EA-85)” (Oxford Biomedical Research, Rochester Hills, MI, USA). The results were expressed in ng 15-F_2t_-IsoP/mg creatinine after normalization with creatinine values. Malondialdehyde was measured using the “QuantiChromTM TBARS Assay Kit (DTBA-100)” (BioAssay Systems LLC, Hayward, CA, USA). For TBARS values > 1.5 µM, the sample was diluted, the assay was performed again, and the result was multiplied by the dilution factor. The results were expressed in relation to creatinine as µmol MDA/mol creatinine. 8-oxodG was quantified with the “DNA Damage (8-OHdG) ELISA Kit (#SKT-120)” (StressMarq Biosciences Inc., Victoria, BC, Canada). The results were related to urine creatinine and expressed in ng 8-oxodG/mg creatinine. Creatinine in urine was determined with the “Creatinine (urinary) Colorimetric Assay Kit (Item No. 500701)” (Cayman Chemical Company, Ann Arbor, MI, USA).

### 2.9. Data Analysis

Metric variables were expressed as median with associated 1st and 3rd quartiles (Q1-Q3) calculated with Tukey pivots, and categorical variables as count with percentages. Since most variables were non-normally distributed (Shapiro–Wilk test), differences and correlations were calculated using nonparametric test procedures.

Group comparisons were done by the Mann–Whitney U test or Wilcoxon signed-rank test and Kruskal–Wallis or Friedman’s test, as appropriate. After applying the Kruskal–Wallis or Friedman’s test, post hoc tests were carried out using Bonferroni correction. Correlations between metric variables were indicated with the rank correlation coefficient according to Spearman (r). Based on Cohen’s classification [25], this measure of effect strength was interpreted as follows: r = 0.10 corresponds to a weak effect, r = 0.30 to a medium effect, and r = 0.50 a strong effect. For nominal variables, the chi-square or Fisher’s exact test was used to test the association. If more than two values were possible for a nominal variable, the Monte Carlo method was used as an exact test.

In addition, covariance (ANCOVA) and regression analyses were carried out for further consideration of group differences and correlations. In order to fulfill the requirements for these parametric methods, it was necessary to log-normalize non-normally distributed variables. The variables transformed with the decadic logarithm were given the prefix “lg”.

Regression analyses were carried out to determine correlations between several explanatory (independent) variables and a dependent variable. The model equation of the multiple linear regression model is:

y = β0 + β1 − x1 + … + βk − xk + ε

Each dependent variable y (e.g., SHp or γ-H2AX) was assigned the independent variable x (e.g., gender or age) which had shown significance in the simple correlation analyses performed previously. The influence of the independent variables is expressed by the standardized coefficient β. The nonstandardized regression coefficient B allows a statement on the quantitative change of y if x changes by one unit. ε corresponds to the residual. The coefficient of determination R2 indicates what percentage of the dependent variable can be explained by the regression coefficients of the model estimation. In the corrected R2, the R2 is adjusted for the number of cases (n) and the number of independent variables.

In this paper, the regression analyses were conducted in an exploratory approach using the backward selection method. The statistical program excluded the independent variable with the highest insignificance (*p* > 0.1) from the model until only those variables remained that made a significant contribution to the explanation of variance. The following dependent variables were assessed: age, sex, BMI, diabetes, sleep apnea, mean systolic blood pressure, renin, eGFR, serum protein, cholesterol, and NT-pro-BNP. The prerequisites for the application of a multiple linear regression model were checked in each case.

In order to examine group differences of a dependent variable more closely, a covariate was added to the model within the framework of an ANCOVA. The quality of the model was also indicated with the corrected R2.

For all statistical procedures, a *p* ≤ 0.05 was recognized as significant. SPSS (version 25, IBM, Armonk, NY, USA) and GraphPad Prism version 6 (GraphPad Software, San Diego, CA, USA) were used for statistical evaluation.

## 3. Results

### 3.1. Study Population

The study participants were divided into three groups according to their intake of antihypertensive drugs and their average systolic blood-pressure values in the ABPM or their practice blood-pressure values (see Table 2). For this purpose, the data from their first visit were used in each case.

Of the total 72 study participants recruited, 8 could be assigned to the control group and 64 to the hypertensive group, with 36 patients showing well-controlled blood pressure and 28 patients showing poorly controlled blood pressure (Table 3).

Table 3 shows that the median age of the controls at the time of the study was significantly lower than the median age of the HypAll and HypWell. Further, significantly fewer individuals in the control group had diabetes mellitus than in the HypAll group. Despite the high diabetes mellitus prevalence of 39.1% among hypertensives, the median HbA1c was in the normal range (see Table 4). The groups of hypertensives showed very similar characteristics, with the following exceptions: in the HypWell group, significantly more individuals than in the HypPoor group had a first diagnosis of a malignant tumor in a period of 16 years to 11 months before the time of the study and had been treated surgically, chemotherapeutically, and/or with radiation therapy. Benign tumors were not included.

In the 24 h urine collection, patients in the HypPoor group more often exhibited proteinuria and albuminuria than the controls and the HypWell individuals. There were no significant group differences in the RAAS parameters. The controls had significantly higher cholesterol levels compared to the hypertensives; only one of them was taking a statin. Other metabolic parameters were similar across groups. As a marker of heart failure, NT-proBNP was significantly higher in the HypPoor and HypAll compared to the controls.

Table 5 shows the systolic blood-pressure values measured during the physical examination of the sitting patient and during the ABPM. According to the group classification, HypPoor had significantly higher systolic blood-pressure values than the controls and HypWell. There were fewer dippers among the HypAll, although not significantly. By far, the most common cause of secondary hypertension was sleep apnea. A total of five study participants had proven primary hyperaldosteronism.

According to the group classification, there were significant differences in the use of antihypertensives between the controls and the hypertensive groups (Table 6). Patients in both the HypWell and HypPoor groups took a median of five antihypertensives. The three most frequently taken substance classes in both groups were calcium antagonists, diuretics, and beta-blockers. ACE inhibitors and AT1R blockers were also taken with similar frequency in both groups. Mineralocorticoid receptor antagonists were taken by 25% in both the HypWell and HypPoor groups. Second-line antihypertensives, such as alpha-1 blockers, tended to be used more frequently in the HypPoor group. Calcium antagonists were taken less frequently in the HypPoor vs. HypWell.

### 3.2. Markers of Oxidative Stress

**Figure 1:**
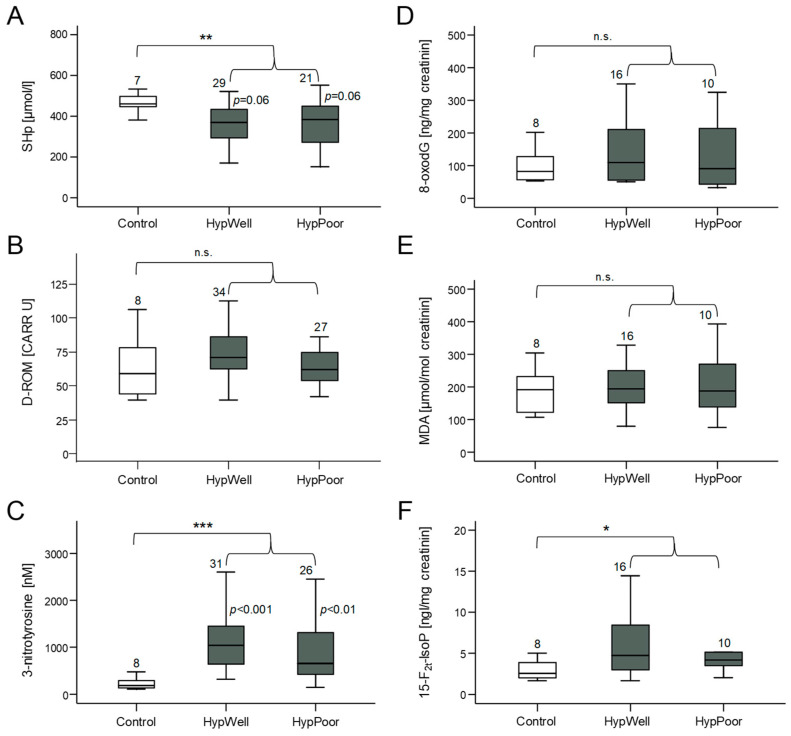
Markers of oxidative stress and antioxidant potential measured in plasma and in urine. (**A**) Amount of SHp (free thiol groups in proteins) as a marker of antioxidant potential in plasma. (**B**) Quantification of D-ROM (derivatives of reactive oxygen species) as a marker of lipid oxidation in plasma. (**C**) Measurement of 3-nitrotyrosine as a marker of nitrosative stress to proteins in plasma. (**D**) Detection of the oxidative DNA base-pair modification 8-oxodG excreted in urine. Quantification of (**E**) MDA (malondialdehyde) and (**F**) 15-F_2t_-IsoP (15-F_2t_-isoprostane) as markers of lipid peroxidation in urine. Shown in each case is the comparison between the control group, HypWell, and HypPoor, with the *p* values inserted next to the standard deviation bar. The brackets indicate the comparison of HypAll to the control. n.s. = nonsignificant, * *p* ≤ 0.05, ** *p* < 0.01, and *** *p* < 0.001. Numbers over the boxplots indicate the number of patient samples analyzed.

Oxidative stress occurs when the formed ROS can no longer be detoxified by the antioxidant capacity of the cells [12]. Then, molecules of the cell are damaged by oxidation. Therefore, we measured markers of this damage (D-ROM, 3-nitrotyrosine, 8-oxodG, MDA, and 15-F_2t_-IsoP) but also a marker of antioxidative capacity (SHp) in both the urine and blood plasma of the study participants. Figure 1 shows that the HypAll group had higher stress levels compared to the control group reflected in a significantly reduced antioxidative capacity in the plasma (SHp) and significantly increased amounts of the protein nitrosylation marker 3-nitrotyrosine and the lipid oxidation marker 15-F_2t_-IsoP. Two other markers of oxidative stress, D-ROM, and 8-oxodG, showed a tendency to increase, whereas MDA did not change. After correcting the group differences for age, this significant difference remained for SHp and 3-nitrotyrosine, with age still showing a significant influence on SHp. For 15-F_2t_-IsoP, the group difference was no longer significant due to the influence of age (see Table 7).

When comparing hypertensive subgroups with the controls, less marked differences were found. SHp was lower in the HypPoor than in the controls, and there was a trend in comparison with the HypWell. High SHp levels indicate less oxidative stress and a higher antioxidative potential. 3-Nitrotyrosine was increased in both the HypWell and HypPoor. It is also noticeable that the HypWell group tended to show higher levels of oxidative stress than the HypPoor group for all markers.

### 3.3. Markers for DNA Damage

**Figure 2:**
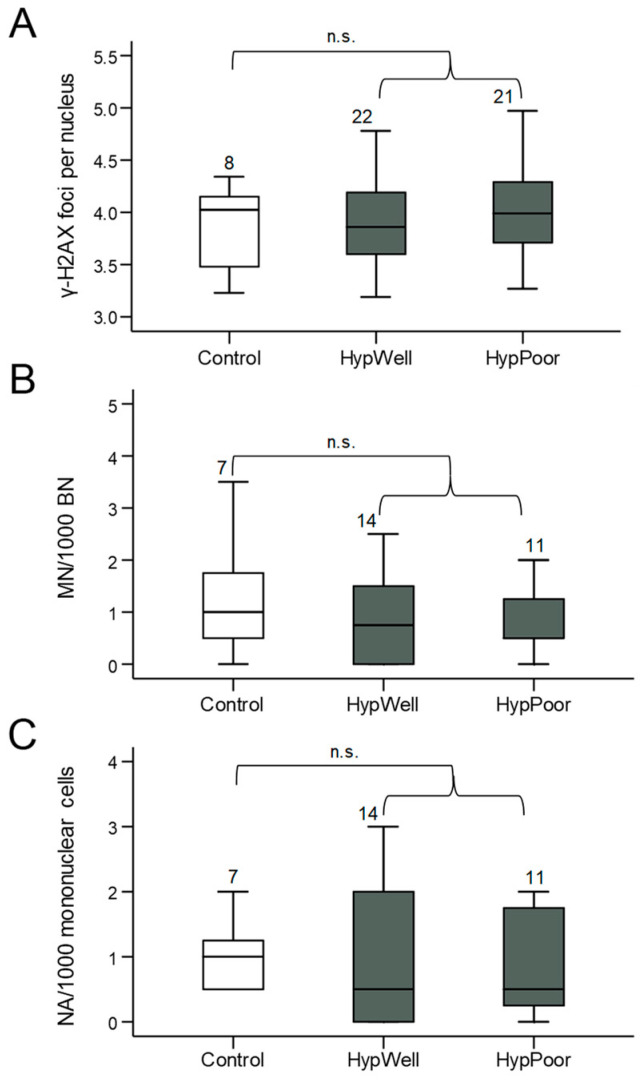
Markers for DNA damage in lymphocytes. (**A**) γ-H2AX foci/nucleus, (**B**) micronuclei/1000 mononuclear cells, and (**C**) nuclear anomalies (NA)/1000 mononuclear cells in lymphocytes isolated from the participants’ blood samples. Shown in each case is the comparison between the control group, HypWell, and HypPoor. The brackets indicate the comparison of HypAll to control. n.s. = nonsignificant. Numbers over the boxplots indicate the number of samples analyzed.

γ-H2AX, as a marker for DNA double-strand breaks and structural DNA damage, and micronuclei, as a marker for chromosomal aberrations, were quantified in lymphocytes of the peripheral blood of the study participants. Anomalies also detectable in the micronucleus frequency test, such as NBUDs and NBLEBs, were not graphically represented individually due to their very low abundance but were shown combined as nuclear anomalies. Using all these markers, no significant differences in DNA damage between the groups could be detected (Figure 2). There was even a tendency for micronuclei to be reduced in the hypertensive patients.

### 3.4. Correlation Analysis

A correlation analysis was performed with the markers for oxidative stress and DNA damage using the clinical parameters collected at the first visit. No correlations with the blood-pressure values were found, except for an inverse correlation between D-ROM and total systolic blood pressure in the ABPM (r = −0.27, *p* ≤ 0.05).

Among the oxidative stress markers themselves, only SHp correlated significantly with 3-nitrotyrosine (r = −0.63, *p* ≤ 0.01). The markers for DNA damage showed no correlations among each other. The only correlation between oxidative stress markers and DNA damage was seen between 3-nitrotyrosine and nuclear anomalies, which was surprisingly negative (r= −0.49, *p* ≤ 0.01).

In the correlation analysis between the markers of oxidative stress or DNA damage with the other metrically scaled clinical parameters, SHp, 3-nitrotyrosine, and 15-F_2t_-IsoP in particular showed effective and significant correlations (Table 8). All three correlated significantly with age, the heart failure marker NT-proBNP, and serum protein. SHp and 3-nitrotyrosine also correlated with renal function parameters, cholesterol, and renin. 8-oxodG showed correlations with metabolic parameters, such as BMI or uric acid. D-ROM and MDA did not show any relevant correlations.

### 3.5. Regression Analyses

In the following, the influencing factors described in Section 3.4 and the parameters of oxidative stress that differed significantly between the groups were examined for their correlation using multiple linear regression (Table 9). Uric acid, although previously shown to have significant correlations with various markers, was excluded from further investigation since no values were available for the control group. CRP and malignancies were also not included in the regression analysis due to their low case numbers.

In the multiple linear regression analysis of SHp, age, sleep apnea, eGFR, and serum protein remained significant influencing variables in the model after backward selection (Table 9). The model itself showed a high model quality (corr. R^2^ = 0.60). Patients with sleep apnea had less SHp. In the backward regression analysis of 3-nitrotyrosine, age, eGFR, and sleep apnea were included as significant influencing factors in the model, whereby patients with sleep apnea had more 3-nitrotyrosine. Age emerged as the only significant influencing factor for 15-F2t-isoprostane (Table 9).

### 3.6. Longitudinal Analysis

Data collection and analysis were performed over a follow-up period for 16 study participants, all of whom were in the HypAll group and whose previous antihypertensive therapy was switched to an adjusted combination therapy. The time between the first visit (V1) and the first follow-up (Fup1) was 7 ± 2 months (16 patients) and between V1 and the second follow-up (Fup2) 14 ± 2 months (11 patients). The range of clinical parameters collected for Fup1 and Fup2 was the same as for V1 (see Table 4). The differences in the metric scaled parameters at the three different time points were tested for significance using the Friedman test. The post hoc test was carried out using the Bonferroni correction. For nominal variables, a constant behavior was postulated, so that they were not considered over time.

Among all the clinical parameters collected, a significant beneficial change over time could only be found for the systolic practice blood pressure measured sitting (Friedman test with *p* ≤ 0.05), accompanied by a similar reduction in ABPM (see Table 10).

The antioxidant potential, measured as SHp, increased significantly over time, reflecting an improved antioxidative defense. This increase occurred from V1 to Fup1. From Fup1 to Fup2, SHp remained constant. This was in contrast to the increase in the two oxidative stress markers D-ROM and 3-NT, which indicate increased oxidative stress. D-ROM decreased from V1 to Fup1 and then increased from Fup1 to Fup2 beyond the baseline value of V1. 3-NT showed a significant increase from both V1 to Fup1 and V1 to Fup2, with the greater increase from V1 to Fup1 (Table 10). The markers for oxidative stress in urine were only determined at one point in time, so that they could not be considered in the longitudinal evaluation.

Regarding γ-H2AX, there were no significant changes over time (Table 10). Nuclear anomalies increased significantly from Fup1 to Fup2. Here, a low number of cases in the parameters of the micronucleus frequency test at V1 have to be considered, which did not allow a comparison with this time point.

## 4. Discussion

This first study of hypertensive patients with well (HypWell) or poorly (HypPoor) controlled blood pressure showed increased systemic oxidative stress but no increased DNA damage compared to healthy individuals. Six different, established markers of oxidative stress to lipids, DNA, and proteins and three markers of DNA damage were quantified, with all markers except one indicating increased oxidative stress in the group of hypertensive patients, three of them significantly. A key strength of this analysis is that the population studied allowed not only for comparison between healthy controls and hypertensive patients but also according to the level of hypertension control. Further, patients were very well characterized for office and ambulatory blood pressures, relevant comorbidities, biomarkers, and medication. Moreover, we were able to perform longitudinal studies on a subset of the hypertensive patients, allowing for the investigation of changes in BP and oxidative markers over 14 months. A major limitation of this study is that, compared with the number of hypertensive patients (n = 64), only a very small and younger control group (n = 8) with normal blood-pressure values could be identified for this analysis; therefore, we also performed age-adjusted and regression analyses of oxidative stress markers.

The two markers analyzed for lipid oxidation in this study, 15-F_2t_-isoprostane and MDA, were measured in urine. 15-F_2t_-isoprostane was significantly elevated in the hypertensive groups, as was reported before in some studies (e.g., [26,27]). Adjustment for age led to a loss of a difference between the hypertensive groups and the controls, as also observed before [28,29]. Two other studies explored the isoprostane values in patients with therapy-resistant hypertension, one finding no difference between individuals with well and poorly controlled hypertension, as we did [30], while the other found significantly elevated isoprostane in therapy-resistant hypertension [31]. Even though not all studies detected significant differences, there, nevertheless, seems to be a positive association between hypertension and isoprostane levels [30,32]. MDA determined in urine was not raised in hypertensive individuals. Although urinary MDA measurement has advantages over serum measurement [33,34], most available data refer to serum-measured MDA, which was elevated in hypertensives. [35,36,37].

The marker for hydroperoxides in lipids, glycosides, or proteins, D-ROM, which was shown to be elevated in cardiovascular and inflammatory diseases, in cancer patients, and also in aging [38,39,40,41], was only tendentially increased in the hypertension groups.

As a measure of antioxidant potential in blood, free thiol groups (SHp) in proteins, which are an important antioxidant force in redox balance and can be used as biomarkers of redox status, were determined photometrically in plasma samples [42,43]. After correction for age, the difference between controls and hypertensives remained significant, although age remained to have a significant effect. Other studies, however, mostly with therapy-naïve hypertensives, also concluded that hypertensives have lower thiol levels than comparable healthy controls [44,45,46,47,48,49]. In contrast, in a study of previously treated hypertensives, no significant differences in thiol levels were found between hypertensives and nonhypertensive control subjects [50]. In the regression analysis, GFR, serum protein, and OSAS were found to influence SHp levels, in addition to age. In the longitudinal study, an increase in SHp of approximately 30% was observed, with a concomitant decrease in blood pressure. Contrary to expectations, HypPoor showed a tendency to slightly higher thiol concentrations than HypWell, which was also found in the study by Tse et al. [51]. In their study, the poorly controlled hypertensives were not only significantly older, for which age adjustment was performed but also took significantly more antihypertensives than the well-controlled hypertensives. So, the antioxidant effect found for some antihypertensives may have been responsible for the difference in thiol levels. In the present study, the HypWell group was on average 4.5 years older than the HypPoor group. Therefore, age fails as an explanation. Both groups were taking the same number of antihypertensive drugs, but calcium channel blockers were less represented in the HypPoor than in the HypWell, so that, in itself, an even worse antioxidant capacity should have been expected in this group, since this class of drugs, like the RAAS blockers, exerts antioxidant effects [52,53].

3-Nitrotyrosine is a marker of nitrosative stress to proteins, and the marker showing the clearest difference between the controls and hypertensives. Elevated 3-nitrotyrosine levels in hypertensives have also been found by other groups [54,55], but this study is the first to show this increase in therapy-resistant hypertensives. Indeed, both subgroups, HypWell and HypPoor, had significantly higher 3-nitrotyrosine levels than the controls. This difference persisted after adjustment for age. As with the other markers, it was notable that HypWell did not have significantly lower levels than HypPoor, possibly due to the age difference, as nitrated proteins accumulate with age as a result of increasingly inefficient proteolysis, as recently reviewed [56]. 3-Nitrotyrosine showed a similar profile of influencing factors as SHp with age, GFR, and OSAS. Increased concentrations of 3-nitrotyrosine have been found in the plasma of patients with decreased renal function [57] and in the plasma of 5/6-nephrectomised rats [58]. Interestingly, in the latter, 3-nitrotyrosine decreased again after administration of the antioxidant tempol. Reduced antioxidant potential has been reported several times for OSAS [59], which may be responsible for the increased 3-nitrotyrosine levels found here. In the longitudinal study, an increase in 3-nitrotyrosine was observed despite a concomitant decrease in blood pressure. Because little is known about the exact degradation and excretion of 3-nitrotyrosine, and only a hesitant change in plasma 3-nitrotyrosine concentrations has been observed [60,61], the time interval between the initial and follow-up examinations was probably not long enough to register treatment-related differences or positive changes were masked by age-related accumulation.

The oxidatively altered nucleic base 8-oxodG acts as a link between oxidative stress and DNA damage markers. As recently reviewed, it reflects the global oxidative stress of an organism, is established as the most important marker of oxidative nucleic base modifications, and is predictive for a number of cancer types [62,63]. Consistent with other studies examining patients receiving antihypertensive therapy [64,65], 8-oxodG was not significantly elevated in the hypertensive patients in our study. In contrast, Subash et al. [66] found elevated 8-oxodG levels in hypertensive patients, with untreated patients having significantly higher levels at diagnosis than patients who had been taking antihypertensive medication for one year. This finding and the results of numerous clinical studies in which a reduction in 8-oxodG was observed with AT1R blocker therapy (e.g., [67,68]), underscore the antioxidant effect of antihypertensive drugs and may explain the lack of a significant difference between the controls and hypertensives that we saw.

The markers discussed up to this point were examined in serum and urine, respectively, and thus reflect oxidative damage to the entire body or, in urine, primarily to the kidney. No markers of oxidative stress were determined in the blood cells themselves, as these would only represent local effects. DNA damage, on the other hand, which will now be discussed, can accumulate in the long-lived lymphocytes [69], where it indicates chronic genotoxic stress and is, therefore, quantified in these cells. Basal DNA damage was measured by staining for γ-H2AX and micronuclei/nuclear anomalies. None of the markers showed increased levels of DNA damage in hypertensives. Similarly, there were no significant differences between subgroups of hypertensives. When the comet assay was used to detect DNA strand breaks, it pointed to an increase in DNA damage in hypertension in vivo, and in human studies [70,71,72,73]. However, when comparing different methods for detecting oxidative DNA damage, there was a broad agreement in the results between the comet assay and other assays, such as the micronucleus frequency test used here [74]. Thus, it can be assumed that increased DNA damage would not have been found with the Comet assay in our patient population either. Moreover, the micronucleus frequency, the only established marker for which a prognostic relevance with regard to tumor risk could be proven so far [17,75], was used. In addition, in vitro, the γ-H2AX assay and the micronucleus frequency test showed a good correlation [76]. Since, in the present study, both tests did not show increased levels of DNA damage in hypertensive patients, the results appear all the more valid. However, this would imply here that the micronucleus test fails as a predictor of increased risk of renal cancer in hypertensive patients. In fact, a recent review shows that micronuclei are more suited for the prognosis of blood, colorectal, and urogenital cancers, but not for, e.g., skin and prostate cancer [17,77]. A better prognosis for urogenital cancers, to which kidney cancers belong, could be achieved by analyzing micronuclei in cells obtained from urine or directly from the affected tissue [78,79].

Furthermore, the patient population investigated in this study differed significantly from that of other studies. Since the patients in the HypAll group were patients with resistant hypertension, this study was preceded by many years of taking various antihypertensive drugs. Thus, in addition to the antioxidant effect of antihypertensive drugs, an antigenotoxic effect can be assumed, which may have prevented an increase in markers for DNA damage in the HypAll group. We have demonstrated this ability of inhibitors of the RAAS, in particular of AT1R blockers and mineralocorticoid receptor antagonists, in vitro and in animal studies [73,80]. This assumption is supported by Subash [71], who found a reduction in DNA damage, measured with the comet assay, after one year of antihypertensive therapy in patients. Additional confirmation is provided by a recent Japanese study, which showed that the risk of dying from various types of cancer was reduced when taking antihypertensives [81]. Furthermore, a large population-based study from Korea found that RAAS blockers better protected against the occurrence of cancer compared to other blood-pressure-lowering drugs [82]. Last but not least, besides their known action in heart failure, RAAS blockers had beneficial effects in preventing cardiotoxicity from antitumor drugs [83].

It would, therefore, be very interesting in a future prospective study, preferably with therapy-naïve patients, to investigate the antioxidative and thus possibly also the antigenotoxic effects of different antihypertensive drugs on oxidative stress, with a focus on RAAS blockers. To distinguish between the effects of individual antihypertensives, it would be desirable to include in this study subjects treated with monotherapy. Since the guideline of the European Society of Cardiology/European Society of Hypertension recommends a combination of two preparations already at the initiation of therapy, except in very mild hypertension [84], it is not possible for us to conduct such a study in Europe.

## 5. Conclusions

In summary, we found increased levels of oxidative stress in treated hypertensive patients, but no increase in basal DNA damage, including a marker known to predict future cancer risk. This suggests that the oxidative stress associated with hypertension in the current study samples was not sufficient to induce measurable DNA damage, which may be due to the antioxidant potential of antihypertensive medication, especially RAAS blockers. Accumulating evidence from in vitro and in vivo experiments, as well as observations from the present study, suggest that the beneficial effects of these compounds might extend beyond the well-described clinical effects of blood-pressure lowering and renal and cardiac protection, and might also protect against oxidative DNA damage. 

## Figures and Tables

**Table 1:** Inclusion criteria for the Best-In-Tension Study and inclusion and exclusion criteria for participants of the MyStIC study.

Inclusion criteria of Best-In-Tension	age over 18 therapy-resistant hypertension signed informed consent
Inclusion criteria of MyStIC	age over 18 signed informed consent
General exclusion criteria of MyStIC	contradictions to undergo cardiac MRI prior cardiac disease ^a^ impaired renal function ^b^ manifest liver disease systemic diseases with cardiac or pulmonary involvement ^c^ pregnancy/lactation
Specific exclusion criteria for healthy volunteers of MyStIC	known arterial hypertension medication that affects blood pressure ^d^ treatment with a mineral corticoid receptor antagonist within the last three weeks

^a^ e.g., atrial fibrillation, myocardial infarction, or coronary artery disease. ^b^ estimated glomerular filtration rate (eGFR) < 30 mL/min. ^c^ e.g., amyloidosis, pulmonary fibrosis. ^d^ e.g., angiotensin I-converting enzyme (ACE) inhibitor/angiotensin II type-1 receptor (AT1R) blocker, calcium antagonist, beta blocker, diuretic, antisympathotonic drug, and alpha-1 blocker. MRI = magnet resonance imaging.

**Table 2:** Definitions applied to study participants.

Group	Subgroup	Antihypertensive Medication	Average Systolic Blood Pressure ^a^
Control		0	<140 mmHg
Hypertensive patients (HypAll)	Well-controlled hypertensives (HypWell)	≥1	<140 mmHg
	Poorly controlled hypertensives (HypPoor)	≥1	≥140 mmHg
		0	≥140 mmHg

^a^ obtained in the ABPM (ambulatory blood-pressure monitoring) or measured in practice.

**Table 3:** Baseline characteristics of the study participants. In addition to ABPM, age, gender, and BMI, previous diseases and lifestyle factors are listed. Shown in each case is the median (Q1–Q3) or the percentage. *p* > 0.05, * *p* ≤ 0.05, ** *p* < 0.01, and *** *p* < 0.001 compared to control, ° *p* ≤ 0.05, °° *p* < 0.01, and °°° *p* < 0.01 compared to HypWell.

	Control (n = 8)	HypWell (n = 36)	HypPoor (n = 28)	HypAll (n = 64)
Systole [mmHg]	126 (122–132)	129 (121–132)	154 (146–163) ***, °°°	137 (128–153) *
Diastole [mmHg]	83 (76–94)	74 (67–77) **	78 (74–92) °°	75 (70–81) *
Age [y]	56.0 (47.5–63.5)	69.5 (62.0–76.0) *	65.0 (59.0–73.0)	66.5 (60.5–73.5) *
Male sex	75%	44.4%	64.3%	53.1%
BMI [kg/m^2^]	27.8 (25.2–28.9)	29.0 (26.4–33.1)	30.4 (28.0–34.0)	29.6 (26.8–33.8) *^p^*^ = 0.07^
Smokers	0%	8.3%	15.4%	11.9%
Diabetes mellitus	0%	33.3% *^p^*^ = 0.08^	46.4% *	39.1% *
eGFR < 60	0%	27.8%	32.1%	29.7%
Chronic inflammatory disease ^a^	0%	8.3%	14.3%	10.9%
Malignancy	0%	16.6%	0% °	9.3%

BMI = body mass index, eGFR = estimated glomerular filtration rate. ^a^ e.g., chronic viral hepatitis and rheumatoid diseases.

**Table 4:** Laboratory parameters of the study participants. The values were all determined from blood with the exception of two values measured in collected urine. The values in bold are above the laboratory reference values for the respective value. Shown in each case is the median (Q1–Q3) or the percentage. *p* > 0.05, * *p* ≤ 0.05, ** *p* < 0.01, *** *p* < 0.001, and *p* > 0.05 compared to control. ° *p* ≤ 0.05, °°° *p* < 0.001 compared to HypWell.

	Control (n = 8)	HypWell (n = 36)	HypPoor (n = 28)	HypAll (n = 64)
**Kidney function**				
eGFR [mL/min/1.73 m^2^]	82 (77–88)	75 (56–86)	77 (57–89)	75 (57–87)
Urea [mg/dL]	23 (23–25)	38 (28–48) **	35 (28–41) **	36 (28–46) ***
Creatinine [mg/dL]	1.01 (0.78–1.06)	0.89 (0.80–1.15)	0.99 (0.77–1.20)	0.96 (0.79–1.17)
**24 h urine collection**				
Proteinuria [mg/d]	71 (39−112)	39 (39–100)	190 (121–237) °	105 (39–216)
Albuminuria [mg/d]	11 (11–11)	11 (11–11)	84 (21–165) **^,^ °°°	11 (11–97) *^p^*^ = 0.06^
**RAAS**				
Renin [ng/L]	7.8 (6.9–10.1)	11.6 (6.5–39.8)	6.2 (2.5–15.4)	9.2 (4.2–28.4)
Aldosterone [ng/L]	45 (38–66)	66 (39–119)	52 (36–101)	58 (37–117)
Aldosterone to renin ratio	5.6 (4.9–8.5)	4.2 (1.2–12.9)	12.0 (2.3–16.4)	7.4 (1.5–16.4)
**Blood parameters**				
HbA_1c_ [%]	5.6 (5.5–5.7)	5.9 (5.6–6.4)	6.0 (5.5–6.6)	5.9 (5.5–6.5)
Cholesterol [mg/dL]	**236** (210–256)	194 (160–221) *	182 (146–224) *	187 (158–221) **
Triglycerides [mg/dL]	142 (114–193)	126 (104–217)	122 (94–205)	124 (98–214)
Uric acid [mg/dL]		5.9 (5.1–7.8)	5.8 (5.1–7.5)	5.8 (5.1–7.8)
Protein [g/dL]	7.2 (6.9–7.5)	7.1 (6.8–7.3)	7.0 (6.8–7.3)	7.0 (6.8–7.3)
NT-proBNP [pg/mL]	41 (30–62)	**103** (48–434) * *^p^*^ = 0.08^	**154** (75–413) *	**109** (59–433) **

eGFR = estimated glomerular filtration rate, HbA_1c_ = glycohaemoglobin, NT-proBNP = N-terminal propeptide of brain natiuretic peptide, RAAS = renin aldosterone angiotensin system.

**Table 5:** Systolic blood-pressure values and secondary forms of hypertension in the study participants. Measurements during the physical examination in the sitting position and during ABPM are shown. Dipping is defined as a night-time drop in blood pressure > 10% of the daily mean. Shown in each case is the median (Q1–Q3) or the percentage. *p* > 0.05, * *p* ≤ 0.05, and *** *p* < 0.001 compared to control. °° *p* < 0.01, and °°° *p* < 0.001 compared to HypWell.

	Control (n = 8)	HypWell (n = 36)	HypPoor (n = 28)	HypAll (n = 64)
**Office measurement**				
Systolic pressure sitting [mmHg]	138 (129–140)	143 (127–152)	154 (148–172) *^,^ °°	149 (136–162) *^p^*^ = 0.09^
**ABPM**				
Mean systolic pressure [mmHg]	126 (123–132)	129 (121–134)	154 (146–160) ***^,^ °°°	136 (128–151) *
Systolic pressure day [mmHg]	133 (125–137)	132 (124–137)	158 (151–166) ***^,^ °°°	140 (129–154) *^p^*^ = 0.07^
Systolic pressure night [mmHg]	118 (114–124)	120 (115–127)	142 (134–157) ***^,^ °°°	131 (119–138) *
Dipper	62.5%	30.6%	35.7%	32.8%
**Secondary causes of hypertension**				
Sleep apnea	0%	30.6%	32.1%	31.3%
Primary hyperaldosteronism	0%	2.8%	7.1%	4.7%
Renal artery stenosis/pheochromocytoma	0%	0%	0%	0%

ABPM = ambulatory blood-pressure monitoring.

**Table 6:** Medication of the study participants. In addition to the antihypertensives taken, the use of statins is shown. The number with percentage share or the median (Q1–Q3) is shown in each case. * *p* ≤ 0.05, *** *p* < 0.001 compared to control. *p* > 0.05 compared to HypWell.

	Control (n = 8)	HypWell (n = 36)	HypPoor (n = 28)	HypAll (n = 64)
ACE inhibitors	0%	50.0% *	39.3% *^p^*^ = 0.08^	45.3% *
AT1R blockers	0%	41.7% *	42.9% *	42.2% *
Aldosterone antagonists	0%	25.0%	25.0%	25.0%
Renin inhibitors	0%	2.8%	3.6%	3.1%
Diuretics	0%	77.8% ***	75.0% ***	76.6% ***
Calcium channel blockers	0%	88.9% ***	67.9% ***^, *p* = 0.06^	79.7% ***
Beta blockers	0%	80.6% ***	71.4% ***	76.6% ***
Alpha-1 blockers	0%	30.6%	50.0% *	39.1% *
Antisympathotonics	0%	38.9% *	32.1%	35.9% *
Direct vasodilators	0%	5.6%	14.3%	9.4%
Number of antihypertensive substance classes	0	5.0 (3.5–6.0) ***	5.0 (2.5–6.0) ***	5.0 (3.0–6.0) ***
Statins	12.5%	44.4%	35.7%	40.6%

ACE = angiotensin I converting enzyme, AT1R = angiotensin II type 1 receptor.

**Table 7:** ANCOVA of individual markers for oxidative stress. For the 3 markers that showed a significant group difference between the control and HypAll, an ANCOVA was performed. The corrected coefficient of determination (R^2^), the number of cases (n), and the significance (*p*) of the model, as well as the significance of the factor group (control vs. HypAll) and the covariate age, are given.

	R^2^	n	*p* (Model)	*p* (Group)	*p* (Age)
SHp	0.31	57	<0.001	0.043	<0.001
3-Nitrotyrosine	0.35	65	<0.001	<0.001	0.148
15-F_2t_-isoprostane	0.26	34	0.003	0.087	0.008

SHp = free thiol groups in proteins.

**Table 8:** Correlation analysis of the markers for oxidative stress, DNA damage, and clinical parameters. All rank correlation coefficients, according to Spearman ≥ 0.20, are shown with the respective number of cases (n) and significance level: * *p* ≤ 0.05, ** *p* < 0.01. eGFR is shown as a representative marker of kidney function, whereby the other parameters of kidney function from blood and collected urine not shown here also correlated significantly.

	Age	eGFR	NT-proBNP	Protein in Serum	BMI	Uric Acid	Cholesterol	Renin
**SHp**	−0.64 ** n = 59	0.49 ** n = 58	−0.52 ** n = 55	0.39 ** n = 57		−0.29 n = 37	0.30 * n = 56	−0.28 * n = 55
**D-ROM**								
**3-NT**	0.37 ** n = 67	−0.45 ** n = 66	0.39 ** n = 61	−0.28 * n = 64		0.36 * n = 44	−0.40 ** n = 64	0.31 * n = 59
**8-oxo-dG**					−0.52 ** n = 34	−0.61 ** n = 20		−0.21 n = 33
**15-F_2t_-IsoP**	0.44 ** n = 34		0.35 * n = 34	−0.39 * n = 34		−0.27 n = 20		
**MDA**	0.26 n = 34		0.33 n = 34		−0.26 n = 34	−0.20 n = 20	0.24 n = 3	
**γ-H2AX**	0.21 n = 51			−0.40 ** n = 51			−0.32 * n = 50	
**MN**						−0.22 n = 21		−0.47 ** n = 32
**NA**				−0.27 n = 32	−0.24 n = 32	−0.31 n = 21	0.25 n = 32	

3-NT = 3-nitrotyrosin, 15-F2t-IsoP = 15-F2t-isoprostane, γ-H2AX = phosphorylated histone 2AX, BMI = body mass index, CRP = C-reactive protein, D-ROM = derivatives of reactive oxygen species, eGFR = estimated glomerular filtration rate, MDA = malondialdehyde, MN = micronuclei, NA = nuclear anomalies, NT-proBNP = N-terminal propeptide of brain natiuretic peptide, and SHp = free thiol groups in proteins.

**Table 9:** Overview of significant interactions of markers of oxidative stress that differ between groups and clinical parameters. Properties of the respective model with corrected coefficient of determination (R2), number of cases (n), and significance (*p*) are shown in the first line. Furthermore, the determined standardized coefficient (β) and significance (*p*) are given for each influencing factor included in the model, where a significant interaction was found. Others are marked with n.s. for nonsignificant.

	SHp Corr. R^2^ = 0.60, n = 36, *p* < 0.001	lg 3-NT Corr. R^2^ = 0.57, n = 38, *p* < 0.001	lg 15-F_2t_-IsoP Corr. R^2^ = 0.22, n = 26, *p* = 0.023
Age	β = −0.45, *p* = 0.002	β = 0.40, *p* = 0.002	β = 0.46, *p* = 0.017
Gender	n.s.	n.s.	n.s.
BMI	n.s.	n.s.	n.s.
Diabetes	n.s.	n.s.	n.s.
Sleep apnea	β = −0.29, *p* = 0.015	β = 0.49, *p* < 0.001	n.s.
Mean systole	n.s.	n.s.	n.s.
Renin	n.s.	n.s.	n.s.
eGFR	β = 0.29, *p* = 0.029	β = −0.33, *p* = 0.007	n.s.
Serum protein	β = 0.34, *p* = 0.007	n.s.	n.s.
Cholesterol	n.s.	n.s.	n.s.
NT-proBNP	n.s.	n.s.	n.s.

3-NT = 3-nitrotyrosin, 15-F2t-IsoP = 15-F2t-isoprostane, BMI = body mass index, eGFR = estimated glomerular filtration rate, SHp = free thiol groups in proteins.

**Table 10:** Systolic blood pressure, markers for oxidative stress in the blood, and markers for DNA damage over time. Fup1 after 7 ± 2 months, Fup2 after 14 ± 2 months. Shown is the median (Q1-Q3) and the number of cases (n). *p* > 0.05, * *p* ≤ 0.05 compared to V1, ° *p* ≤ 0.05, °° *p* < 0.01 compared to Fup1.

	V1 (n = 16)	Fup1 (n = 16)	Fup2 (n = 11)
**Measurement in practice**			
Systolic pressure sitting [mmHg]	152 (144–161)	133 (131–150)	135 (122–148) *
**ABPM**			
Mean systolic pressure [mmHg]	137 (130–157)	136 (128–142)	130 (128–149)
Systolic pressure day [mmHg]	146 (134–162)	140 (131–147)	135 (133–154)
Systolic pressure night [mmHg]	129 (120–146)	125 (120–129)	119 (115–128)
**Markers of oxidative stress**			
SHp [µmol/L]	294 (260–370)	379 (295–478)	379 (347–457) *
D-ROM [CARR U]	64 (55–70)	59 (46–69)	70 (61–80) *^p^*^ = 0.07,^ °°
3-NT [nM]	966 (671–1311)	1032 (699–2061) *	1072 (685–1452) *
**Markers of DNA damage**			
γ-H2AX-Foci/cell	3.81 (3.69–4.20)	3.90 (3.73–4.13)	4.23 (3.90–4.38)
MN/1000 mononuclear cells	0.75 (0.00–1.50)	1.00 (0.50–1.75)	1.00 (0.50–1.50)
NA/1000 mononuclear cells	1.00 (0.00–2.00)	0.50 (0.00–1.75)	1.50 (0.50–2.00) °

Fup = follow-up, 3-NT = 3-nitrotyrosin, γ-H2AX = phosphorylated histone 2AX, ABPM = ambulatory blood-pressure monitoring, D-ROM = derivatives of reactive oxygen species, MN = micronuclei, NA = nuclear anomalies, SHp = free thiol groups in proteins.

## Data Availability

The data presented in this study are available on request from the corresponding author. The data are not publicly available due to privacy reasons.
